# Exploring the Mystery of the Tetrahydrobiopterin Synthetic Defect Lethal Mutant *lem^l^* from Birth to Death in the Silkworm *Bombyx mori*

**DOI:** 10.3390/ijms232012083

**Published:** 2022-10-11

**Authors:** Dan Liang, Rui Shu, Song Jiang, Mengjun Xu, Yangyang Cai, Hongwei Qin, Daobo Zhang, Mengwei Feng, Junshan Gao, Yan Meng

**Affiliations:** 1 School of Life Sciences, Anhui Agricultural University, 130 West Changjiang Road, Hefei 230036, China; 2 Institute of Sericulture, Anhui Academy of Agricultural Sciences, 15 Huoshan Road, Hefei 230061, China; 3 Anhui International Joint Research and Development Center of Sericulture Resources Utilization, Hefei 230036, China

**Keywords:** maternal effect, mitochondrial damage, immune system, apoptosis system, oxidative stress

## Abstract

Tetrahydrobiopterin (BH4) is a vital coenzyme for several enzymes involved in diverse enzymatic reactions in animals, and BH4 deficiency can lead to metabolic and neurological disorders due to dysfunction in its metabolism. In the silkworm natural homozygous mutant *lem^l^*, the key enzyme sepiapterin reductase (BmSPR) in the de novo synthesis pathway of BH4 is inactivated, resulting in severe deficiency of BH4 synthesis. However, it is not known why the *lem^l^* larvae can survive to the second-instar stage and which pathways lead to their death when BH4 is deficient. Here, we quantified BH4 and found that the fertilized eggs contained large amounts of BH4 transferred from the mother to the offspring, maintaining its normal development in the embryo and the first instar. Subsequently, we investigated the multiple pathways in which BH4 is involved as a cofactor. The results showed that BH4 deficiency in silkworms blocked the melanin synthesis pathway, caused an insufficient degree of epidermal sclerosis, disordered tyrosine metabolism, and damaged mitochondria. On the other hand, BH4 deficiency led to the uncoupling of nitric oxide synthase (BmNOS), a reduced NO production, and a significantly reduced fat in fat body catalyzation by phospholipase A2, resulting in an impaired immune system. Meanwhile, the uncoupling of BmNOS increased the O_2_^−^ content, damaged the DNA, and caused the apoptosis of the body cells. Taken together, BH4 is critical for the life and death of *lem^l^* mutants. This study lays a foundation for the further exploration of lepidopteran insects and provides an important basis for the treatment of human BH4 deficiency-related diseases.

## 1. Introduction

There are many natural mutants of silkworm, such as *so*, *ch*, and *mln* [1,2], which can be used as disease research models in nature. Among them, luteal color (*lem*) and luteal color lethal (*lem^l^*) mutants are models associated with tetrahydrobiopterin (BH4) deficiency. The *lem^l^* mutant is normal in embryonic development and the first instar. After the first molting, the silkworm’s body turns bright yellow, and the silkworm stops eating. The head of the silkworm sways frequently until it dies. Meng et al. [3] have demonstrated that the death of *lem^l^* is due to the inactivation of sepiapterin reductase (SPR, EC1.1.1.153), a key enzyme in the BH4 de novo synthesis pathway of *Bombyx mori*, resulting in a severe deficiency of BH4 synthesis. Tsujita et al. [4] found that a yellow pterin in *lem^l^* silkworms has a maternal effect, and we therefore hypothesize that the BH4 required for embryonic and first-instar development can only be derived from fertilized eggs, and there may be a maternal effect. However, it is not yet known why *B. mori* can survive to the second instar and which pathways lead to its death when BH4 synthesis is severely insufficient.

The BH4 is an essential coenzyme for aromatic amino acid hydroxylases, such as phenylalanine hydroxylase (PAH, EC1.14.16.1), tyrosine hydroxylase (TH, EC1.14.16.2), and tryptophan hydroxylase (TPH, EC1.14.16.4). At the same time, it is also an important cofactor of nitric oxide synthase (NOS, EC1.14.13.39) [5]. These enzymes play important roles in the metabolism of amino acids and the biosynthesis of monoamine neurotransmitters [6]. When BH4 is insufficient, tyrosine metabolism is abnormal, and the TH-catalyzed tyrosine production to dopa is blocked [7], which is necessary for the pigmentation and hardening of the insect epidermis [8]. On the other hand, NOS is the key enzyme that catalyzes the generation of nitric oxide (NO). Studies in mammals have shown that when NOS is uncoupled in the absence of BH4, NO cannot be produced normally, and superoxide anion (O_2_^−^) is generated [9]. However, it remains to be further studied whether BH4 deficiency in *B. mori* causes changes in these pathways.

NO is an important active substance in eukaryotic cells and plays a crucial role in physiological processes such as vasodilation, nerve signaling, and immunity [10]. In *Spodoptera exigua*, NO can activate the activity of phospholipase A2 (PLA2, EC3.1.1.4), a key enzyme in fat synthesis, to synthesize eicosanoids and mediate the expression of antimicrobial peptide (AMP) genes [11]. Insufficient NO synthesis may cause the dynamin-related protein 1 (DRP1) nitrosolate levels to decrease [12] and fail to initiate the normal process of mitochondrial division, resulting in excessive fusion. Mitochondrial damage can release pro-apoptotic substances [13] such as cytochrome C (CytC) [14]. On the other hand, excess O_2_^−^ can lead to DNA fragmentation, protein damage, and biofilm oxidation, further prompting apoptosis [15]. Therefore, due to the severe deficiency of the important cofactor BH4 in *lem^l^* homozygotes, the changes in amino acid metabolism, melanin metabolism, mitochondrial function, lipid synthesis function, immune system function, and apoptosis system function in *lem^l^* homozygotes should be further studied.

Based on previous studies, we know that BmSPR inactivation in *lem^l^* homozygotes leads to severe BH4 deficiency. Therefore, in this study, we took the natural mutant *lem^l^* as the research object to explore the effects of BH4 on silkworm growth and conducted in-depth research on the melanin metabolism pathway, amino acid metabolism pathway, immune system, and apoptosis system that BH4 was involved in as a cofactor, with the aim to uncover the mystery of its life and death. This accelerates the process of basic research on BH4 metabolism, further improves the understanding of metabolic regulation mechanism in *B. mori*, and provides a theoretical basis for the application of BH4 in the treatment of related diseases.

## 2. Results

### 2.1. The Fertilized Eggs Contain a Large Amount of BH4 Transferred from the Mother

**Figure 1:**
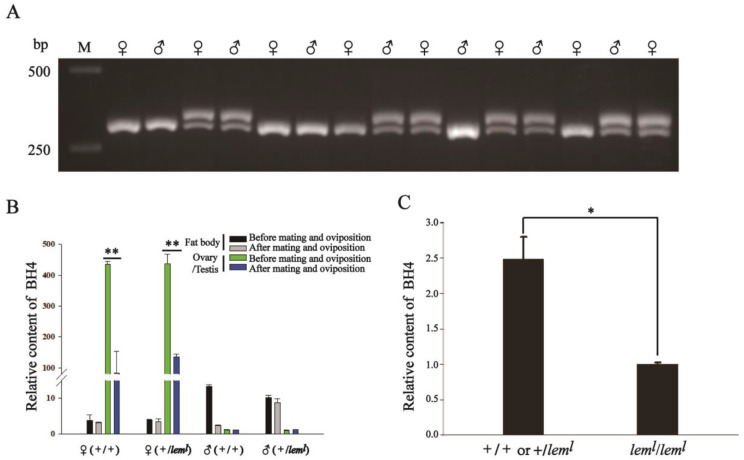
BH4 was transferred to fertilized eggs after oviposition in *lem^l^* heterozygous female moths. (**A**) Genotypic identification of genomic DNA in *lem^l^* moth. M: marker, the bottom one is 250 bp, the top one is 500 bp. ♀ represents the female moth, ♂ represents the male moth. The lane with a single band indicates homozygotes with genotype +/+, and the lane with two bands indicates heterozygotes with genotype +/*lem^l^*. (**B**) The relative content of BH4 in fat body and gonads of *lem^l^* heterozygous moth. (**C**) The relative content of BH4 in *lem^l^* second-instar silkworms. Error bars represent mean ± SD; * *p* < 0.05; ** *p* < 0.01.

In the case that BmSPR inactivation in *lem^l^* homozygotes could not synthesize BH4, we believe that BH4 required to ensure that embryo and first instar can develop properly can only be obtained from fertilized eggs. To explore the reasons for the normal survival of *lem^l^* homozygous lethal mutants before the second instar, we first identified the genotypes of genomic DNA in *lem^l^* moths. The results, as shown in Figure 1A, indicate that lanes with single bands represented wild type moths (+/+), whereas lanes with two bands showed *lem^l^* heterozygous moths (+/*lem^l^*), because the *BmSpr* sequence in the *lem^l^* mutant has a continuous insertion of 27 nt compared with the normal type *BmSpr* sequence (Figure S1). Subsequently, the HPLC detection results demonstrated that the content of BH4 in the gonads of wild-type female and *lem^l^* heterozygous female moths decreased significantly after mating and oviposition (Figure 1B). Additionally, the detection results of the BH4 content in the second-instar larvae showed that the BH4 content in *lem^l^* homozygotes was significantly lower than that in wild-type silkworms (+/+ or +/*lem^l^*) (Figure 1C). These indicate that the fertilized eggs produced by female moths after mating and oviposition contain a large amount of BH4 transferred from the mother.

### 2.2. The Disorder of Tyrosine Metabolism Results in the Damage of Mitochondrial Structure and Function

**Figure 2:**
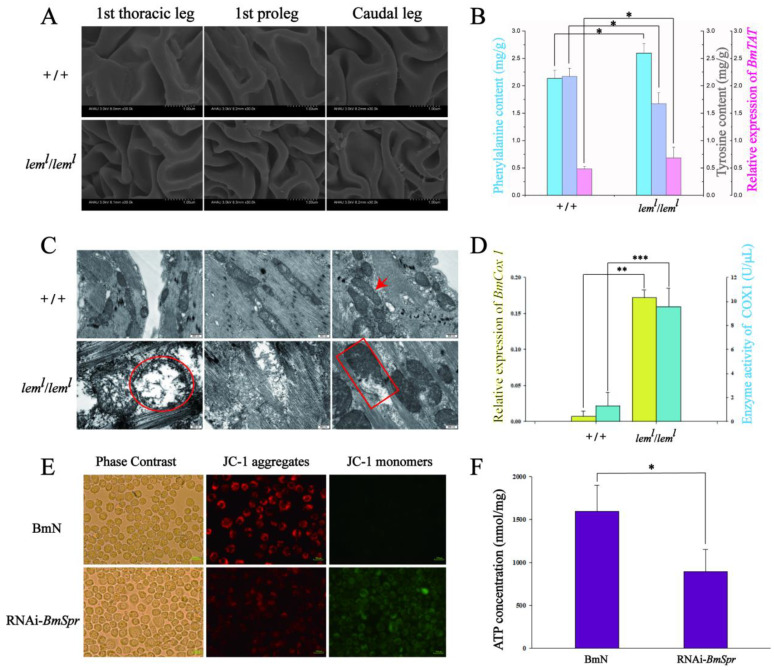
Abnormal amino acid metabolism in *lem^l^* homozygotes led to mitochondrial damage. (**A**) Scanning electron microscope of *lem^l^* epidermis at 30,000× magnification. (**B**) The content of tyrosine (represented by blue columns) decreased, the content of phenylalanine (represented by purple columns) and the expression of *BmTAT* (represented by pink columns) increased in *lem^l^*. (**C**) Transmission electron microscopy of mitochondria in the head of *lem^l^*. The three panels under the same strain represent three different perspectives. The red arrow represents normal mitochondrial morphology, the red circle represents obvious vacuoles in swollen mitochondria, and the red rectangle represents rupture of swollen mitochondrial membrane and outflow of mitochondrial matrix. The scale bar in the lower right corner represents 500 nm. (**D**) The gene expression level (represented by yellow columns) and enzyme activity (represented by blue columns) of COX1 increased in *lem^l^*. Mitochondrial membrane potential (**E**) and ATP content (**F**) decreased after RNAi-*BmSpr* in BmN cells. The scale bar in the lower right corner represents 200 μm. JC-1 is an ideal fluorescent probe widely used to detect mitochondrial membrane potential. At low mitochondrial membrane potential, JC-1 is predominantly a monomer that yields green fluorescence with emission of 530 ± 15 nm. At high mitochondrial membrane potential, the dye aggregates yielding a red- to orange-colored emission (590 ± 17.5 nm). All silkworm body materials were from second-instar *lem^l^* homozygous mutants. The accession numbers of genes and proteins involved in the figure are shown in Table 1. Data are mean values ± S.E.M (*n* = 3). Error bars represent mean ± SD; * *p* < 0.05; ** *p* < 0.01; *** *p* < 0.001.

In the process of feeding, we found that the body of *lem^l^* homozygous became soft at the second instar, and its epidermal structure was therefore observed by scanning electron microscopy (SEM). The results indicate that the epidermis of lethal individuals had more folds than that of the wild-type individuals at the second instar (Figure 2A), and the degree of sclerosis was insufficient. Therefore, we speculated that the gradual death of *lem^l^* homozygotes from the second instar was related to the melanin metabolic pathway or amino acid metabolism using BH4 as a cofactor.

To verify the conjecture, we first detected the amino acid content involved in tyrosine metabolism in *lem^l^* homozygotes. Compared with the wild-type silkworm, the phenylalanine content of the second-instar *lem^l^* homozygous lethal type silkworm was significantly increased, the tyrosine content was significantly decreased, and the gene level of *tyrosine aminotransferase* (*BmTAT*) was significantly increased (Figure 2B). These findings indicate that the tyrosine metabolism in *lem^l^* lethal silkworms was disordered. In view of the phenomenon that the head of the second-instar *lem^l^* homozygote frequently swayed, we observed the status of mitochondria by transmission electron microscopy (TEM). Compared with the wild-type silkworm, the mitochondria in the head of *lem^l^* homozygous mutant showed obvious swelling, a large number of cristae were broken, a small number of unfractured cristae were disordered, the membrane boundary was also broken and blurred, the mitochondrial matrix flowed out, and there was obvious damage (Figure 2C). The related detection results of the mitochondrial self-coding gene *cytochrome c oxidase subunit 1* (*COX1*) showed that BmCOX1 gene expression level and enzyme activity were significantly increased in *lem^l^* homozygous lethal individuals at the second instar (Figure 2D). In addition, to simplify the research background and more accurately study the situation of mitochondria when BH4 synthesis is insufficient, we constructed an RNAi-*BmSpr* model in silkworm ovary cells (BmN). The qRT-PCR results indicated that the interference model was successfully constructed (Figure S2). The results of the intracellular interference experiments revealed that the membrane potential and ATP content of RNAi-*BmSpr* cells were significantly decreased compared with normal BmN cells (Figure 2E,F). The above results prove that the deficiency of BH4 in *B. mori* will block the melanin synthesis pathway and lead to insufficient degree of epidermal sclerosis, disorder of tyrosine metabolism, and obvious damage to mitochondria.

### 2.3. NO Reduction after NOS Uncoupling Leads to an Impaired Immune System

**Figure 3:**
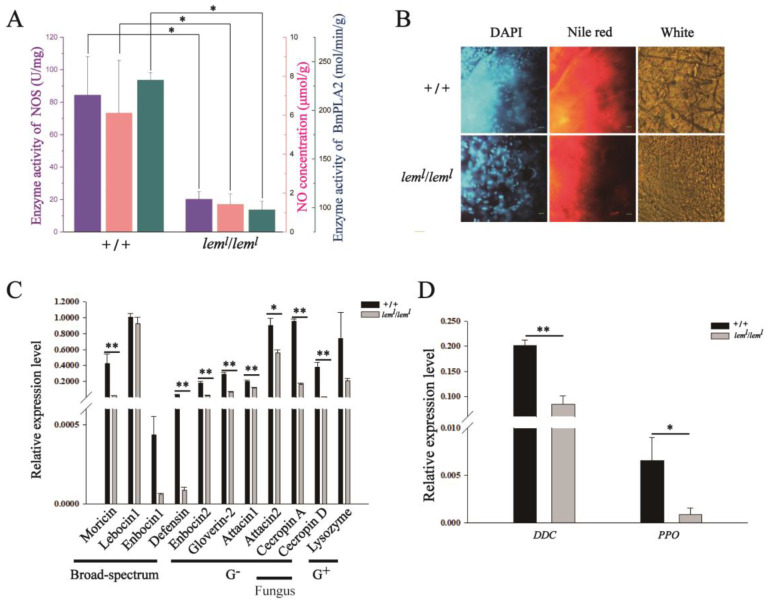
Decrease in NOS enzyme activity led to the decrease of NO, which damaged the immune system in *lem^l^* homozygotes. (**A**) NOS (represented by blue columns) and PLA2 enzyme activities (represented by pink columns) decreased, and NO content (represented by green columns) decreased in *lem^l^*. (**B**) The content of liposomal lipids in *lem^l^*. The scale bar in the lower right corner represents 200 μm. (**C**) The expression level of antimicrobial peptides in *lem^l^*. The detected genes included anti-Galanz positive bacteria gene, anti-Galanz negative bacteria genes, anti-broad-spectrum antibacterial genes and anti-fungal genes, which were represented by G^+^, G^−^, broad-spectrum and fungus in the figure, respectively. (**D**) Expression level of cellular immune genes in *lem^l^*. All materials were from second-instar *lem^l^* homozygous mutants. The accession numbers of genes and proteins involved in the figure are shown in Table 1. Data are mean values ± S.E.M (*n* = 3). Error bars represent mean ± SD; * *p* < 0.05; ** *p* < 0.01.

According to transcription level analysis, BmNOS1 was mainly expressed in silkworms (Figure S3). The detection results of enzyme activity and NO contents in silkworms demonstrated that the enzyme activities of NOS and PLA2 and the NO content in the *lem^l^* homozygous mutant were significantly decreased compared to those of wild-type silkworms (Figure 3A). The NOS enzymatic activity and NO content were also significantly reduced in BmN cells after RNAi-*BmSpr* compared with normal BmN cells (Figure S4A,B). To further verify the function of NOS, BmN cells were incubated with the NOS inhibitor NG-nitro-L-arginine methyl ester (L-NAME). After 24 h, the detection results revealed that the content of NO decreased with the increase of inhibitor concentration in a dose-dependent manner (Figure S4C). The above three aspects can be explained as follows: when the synthesis of BH4 is insufficient, the uncoupling of NOS in the silkworm reduces the production of NO. In addition, to determine the effect of NO concentration changes on cell proliferation ability, we added the NO supplement isosorbide mononitrate (ISMN) at a concentration of 0.1 mM to the BmN cells of RNAi-*BmSpr*, which significantly improved the proliferation ability of RNAi-*BmSpr* cells after incubation for 12 and 36 h (Figure S4D). This further illustrates that the reduction of NO in BmN cells may lead to the reduction of cell proliferation ability.

The staining results showed that the lipid content in the fat body of the second-instar *lem^l^* was significantly lower than that of the wild-type silkworms (Figure 3B). The results of qRT-PCR indicated that the expression levels of major antibacterial peptides (AMPs) and cellular immunity genes in the second-instar *lem^l^* homozygotes all decreased compared with the wild-type *B. mori*, among which the expression levels of *Moricin*, *Enbocin2*, *Defensin*, *Gloverin2*, *Attacin1*, *Attacin2*, *Cecropin A*, *Cecropin D*, and two key cellular immunity genes, *dopa decarboxylase* (*DDC*) and *polyphenol oxidase* (*PPO*), were significantly down-regulated (Figure 3C,D). The results of the antibacterial activity test revealed that the colonies grown after the wild-type silkworm body fluids had been coated with *S. aureus* and *E. coli* plates were significantly less than those after coating with *lem^l^* body fluids (Figure S5). This indicates that the antibacterial ability of *lem^l^* body fluid was weaker than that of wild-type silkworm in vitro. These results suggest that NOS uncoupling and NO production are reduced due to BH4 deficiency in silkworms, and fat synthesis catalyzed by PLA2 decreases significantly, resulting in impaired immune system functioning.

### 2.4. Increased O_2_^−^ Leads to the Activation of the Apoptosis System

**Figure 4:**
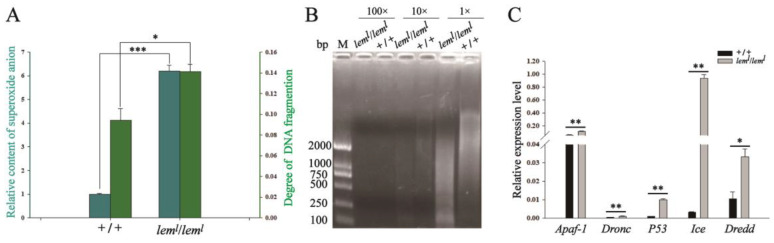
Increase in O_2_^−^ led to DNA fragmentation and started the apoptotic system in *lem^l^* homozygotes. (**A**) The content of O_2_^−^ (represented by dark green columns) and the proportion of somatic DNA fragmentation (represented by light green columns) increased in *lem^l^*. (**B**) The gel electrophoresis detection of *lem^l^* somatic cell DNA fragmentation. 1×: undiluted sample, 10×: diluted 10 times, 100×: diluted 100 times. (**C**) The expression level of pro-apoptotic gene increased in *lem^l^*. All silkworm body materials were from second-instar *lem^l^* homozygous mutants. The accession numbers of genes and proteins involved in the figure are shown in Table 2. Data are mean values ± S.E.M (*n* = 3). Error bars represent mean ± SD; * *p* < 0.05; ** *p* < 0.01; *** *p* < 0.001.

Studies in other species have shown that a decrease in NO synthesis is accompanied by an increase in O_2_^−^ production after NOS uncoupling. Therefore, after knowing that the deficiency of BH4 leads to NOS uncoupling, we examined the O_2_^−^ level in *lem^l^* homozygotes. The results demonstrated that the O_2_^−^ content in the *lem^l^* homozygous mutant was significantly increased compared with the wild-type silkworms (Figure 4A). The O_2_^−^ content in BmN cells with RNAi-*BmSpr* was also significantly increased compared with normal BmN cells (Figure S6A). After the addition of L-NAME in normal BmN cells, the content of O_2_^−^ increased in a dose-dependent manner (Figure S6B). The above three aspects indicate that when the BH4 synthesis in silkworms is insufficient, the uncoupling of NOS increases the production of O_2_^−^. In addition, to determine the effect of O_2_^−^ concentration changes on cell proliferation ability, we added the O_2_^−^ remover superoxide dismutase (SOD) at a concentration of 50 U/mg to the BmN cells of RNAi-*BmSpr*, which significantly improved the proliferation ability of RNAi-*BmSpr* cells after incubation for 12, 36, and72 h (Figure S6C). This further illustrates that the increase of O_2_^−^ in BmN cells may lead to the reduction in cell proliferation ability.

Subsequently, we extracted the DNA of *lem^l^* and wild-type silkworms by the diphenylamine method. The results of the analysis showed that the proportion of DNA fragmentation in the second-instar *lem^l^* homozygotes was significantly increased compared with wild-type silkworms (Figure 4A). The AGE results revealed that the DNA fragments of the second-instar *lem^l^* homozygotes were mainly concentrated in the small fragment region below 2000 bp, and the DNA fragments of the second-instar wild-type silkworms were mainly concentrated in the large fragment region above 2000 bp (Figure 4B). This leads us to infer that the DNA was severely fragmented due to the increase of O_2_^−^ in *lem^l^* homozygotes. The results of qRT-PCR showed that the expression levels of the pro-apoptotic genes *Apaf*, *Dronc*, *P53*, *Ice*, and *Dredd* in *lem^l^* homozygotes were significantly increased compared with wild-type silkworms (Figure 4C), indicating that when BH4 synthesis is insufficient, NOS uncoupling can increase the content of O_2_^−^, damage DNA, and induce apoptosis.

## 3. Discussion

*Bombyx mori* is the most suitable model organism for the identification of disease-related genes among lepidopteran insects, mainly because its genome sequence and high-density linkage map have been published [16,17], and because of the existence of numerous mutants in nature. Further research on silkworms can provide a reference for the treatment of related diseases in other animals. Among them, the mutant *lem^l^* is considered as a potential insect model of human SPR deficiency [3] and is often used to study the function of BH4, but the metabolic mechanism of the mutant in vivo regulating life and death remains unknown.

As early as 1963, through reciprocal cross and ovarian transplantation experiments, it was concluded that the relative content of yellow pigment in *lem^l^* was affected by the mother [4]. Therefore, *lem^l^* homozygous mutant larvae could normally live to the second instar, even though the BmSPR enzyme activity of the *lem^l^* homozygous mutant was defective and could not synthesize BH4, which gave us reason to speculate that BH4 in the *lem^l^* homozygous mutant came from the female moth. The results suggest that BH4 in the ovary of *lem^l^* heterozygous female moths decreased significantly after mating and oviposition (Figure 1B), indicating that a large amount of BH4 in the ovary entered the fertilized eggs and transferred BH4 to the offspring through maternal influence for the growth and development of the embryo and the first-instar larvae. This maternal effect is also present in other species. For example, Lu et al. found it in C57/BL6J mice, showing that maternal probiotic supplementation may improve neurological outcomes in the offspring [18]. Another study revealed that the female beewolf grows helpful bacteria inside her antennae and transfers some to her young. The bacteria produce antibiotics that protect the larvae and their cocoons from mold [19]. A similar phenomenon occurs in plants: the genotype of the maternal plant usually controls the color of the seed coat, and the embryonal control of seed coat color is related with phenylalanine and alanine metabolism in the embryo [20].

Recently, studies on the relationship between amino acids and lifespan have attracted the attention of many scholars. A variety of amino acids have been found to delay aging and prolong life [21]. The latest research claims that BH4 treatment can influence tyrosine synthesis and help to improve outcomes of phenylketonuria patients [22]. Therefore, as a cofactor of hydroxylase of various aromatic amino acids, it is unclear whether the deficiency of BH4 causes the disorder of amino acid metabolism in silkworms and is the cause of death of the *lem^l^* homozygote. Based on this, we detected the contents of relevant amino acids and enzymes. The results suggested that when BH4 was insufficient, the content of tyrosine converted from phenylalanine decreased significantly (Figure 2B). Meanwhile, the expression level of *BmTAT* was significantly increased (Figure 2B), which activated the tyrosine degradation pathway, leading to a significantly lower tyrosine content in the *lem^l^* homozygote compared with wild-type silkworms. The accumulation of phenylalanine in silkworms also hindered the synthesis of tyrosine from the source, resulting in the decrease in the tyrosine content and the disorder of amino acid metabolism. At 48 h after adding 0.2 mM of tyrosine to BmN cells of RNAi-*BmSpr*, the levels of lipid peroxidation and reactive oxygen species were detected. Based on the results, the addition of appropriate amounts of tyrosine could improve the damage degree of BmN cells caused by BH4 deficiency (Figure S7). In addition, the survival time of *lem^l^* homozygotes can be significantly prolonged by feeding with appropriate tyrosine levels from the first instar (data not available). This is consistent with the results of previous studies reporting that dopamine supplementation can save *lem^l^* vitality [3] because dopamine is the product of the melanin metabolic pathway, where tyrosine resides. On the other hand, the melanin metabolic pathway, where tyrosine is located, is involved in the innate immune process and the formation of the exoskeleton in insects [23]. Studies have shown that insufficient sclerosis of the silkworm’s epidermis, especially at the mandible, can cause the *al* and *lem^l^* lethal silkworms to be unable to chew mulberry leaves [24]. In this study, it was obvious that the second-instar *lem^l^* homozygotes had more epidermal folds and insufficient sclerosis at the first thoracic leg, first proleg, and caudal leg compared with wild-type silkworms (Figure 2A). In conclusion, the melanin metabolic pathway is abnormal when the silkworm BH4 is deficient. Combined with the two factors, the decrease in the tyrosine level is one of the reasons for the shortened lifespan of silkworms.

Based on phenylketonuria genetic mice, rats submitted to different hypothalamic-pituitary-adrenal models, and cell cultures exposed to phenylalanine, there is growing evidence that high levels of this amino acid impair mitochondrial bioenergetics and provoke changes in oxidative and inflammatory status [25]. Bailey et al. found that in murine endothelial cells, the reduction of BH4 levels changed the mitochondrial metabolism/bioenergetic balance and inhibited mitochondrial division [26], thereby increasing the size of individual mitochondria. In this study, the mitochondria in the head tissues of the second-instar *lem^l^* homozygote were significantly swollen, with the rupture of the membrane structure and the outflow of matrix (Figure 2C). The membrane potential and ATP content in BmN cells were significantly decreased after RNAi-*BmSpr* (Figure 2E,F). These results indicate that the mitochondrial damage in silkworms is obvious in the absence of BH4. In addition, mitochondrial self-coding gene COX1 had cyclooxygenase and catalase activities. In the second-instar *lem^l^* homozygotes, the expression level and enzyme activity of COX1 were significantly increased compared with wild-type silkworms (Figure 2D), indicating that mitochondrial damage can lead to a stronger oxidative stress response.

The BH4 is not only a coenzyme of aromatic amino acid hydroxylase but also an important cofactor of NOS. When BH4 is deficient, NOS uncouples to produce O_2_^−^ in place of NO [10]; O_2_^−^ is a strong oxide that causes irreversible damage to proteins and DNA. Studies have found that lipopolysaccharide (LPS) stimulation induces the expression of NOS in *B. mori*, and the addition of L-NAME into *Antheraea pernyi* reduced the expression of AMPs [27,28]. This suggests that NOS is correlated with the immune system of silkworms. Moreover Sadekuzzaman et al. [29], in *Spodoptera exigua*, showed that NO activates PLA2 and eicosanoid biosynthesis, which ultimately mediates various immune responses. Therefore, how NOS affects the immune system in the silkworm becomes a new target for our exploration.

In this study, the decreased NOS enzyme activity and NO production in *lem^l^* homozygotes resulted in a decreased enzyme activity of downstream PLA2 (Figure 3A), a decreased lipid content (Figure 3B), and a significantly down-regulated AMP gene expression (Figure 3C,D), which affected the immune system. On the other hand, increased O_2_^−^ production (Figure 4A) led to DNA fragmentation (Figure 4A,B), and the expression of apoptosis-related genes was significantly up-regulated (Figure 4C), causing apoptosis. These results suggest that BH4 deficiency in silkworms directly decreases the activity of NOS, which requires it as a cofactor, and indirectly decreases immune function and proliferative capacity. It also indicates that the homeostasis levels of NO and O_2_^−^ are crucial for the survival of *B. mori*.

Furthermore, BH4 is a cofactor of TPH in addition to the above-mentioned PAH, TH, and NOS. The TPH induces a highly specific catalytic reaction that converts L-tryptophan (tryptophan) to 5-hydroxy-L-tryptophan (5-HTP), which is subsequently used as a substrate by aromatic L-amino acid decarboxylase (DDC) to form 5-hydroxy tryptamine (5-HT). The 5-HT is an ancient intracellular signaling molecule that is widely distributed in the animal kingdom and involved in regulating the behaviors of animals with nervous systems [30]. However, the study of Meng et al. [3] manifested that serotonin administration did not show any positive effects on survival rates and feeding behavior of the *lem^l^* larvae, and we therefore did not explore this pathway in depth.

To sum up, in the case that BmSPR enzyme activity of *lem^l^* homozygotes is deficient and BH4 cannot be synthesized, BH4 is transferred to the offspring by the mother after mating and oviposition to ensure the normal survival of *lem^l^* homozygotes in the first stage. Subsequently, when BH4 is seriously deficient in *lem^l^* homozygotes, amino acid metabolism is disordered, mitochondria are damaged, oxidative stress response in vivo is intensified, the immune system is damaged, and the apoptosis system is activated, which eventually leads to the death of *lem^l^* homozygotes. This highlights the importance of BH4 in maintaining the normal growth and development of silkworms and is consistent with the findings that BH4 deficiency in mammals can cause diseases such as phenylketonuria, Parkinson’s syndrome, and atherosclerosis [31,32]. At this point, we have completely uncovered the life and death mystery of *lem^l^* homozygotes. This study further clarified the source of BH4 and a series of downstream reactions caused by its deficiency, which provides an important basis for the treatment of human BH4 deficiency-related diseases.

## 4. Materials and Methods

### 4.1. Silkworms, Plasmids, and Cell Strains

The silkworm larvae of wild-type strain a65 (+/+ or +/*lem^l^*) and luteal homozygous mutant strain a65 (*lem^l^*/*lem^l^*) were routinely maintained in our laboratory and fed fresh mulberry leaves and maintained at 25 °C [33]. The interference plasmids *BmSpr*-PXL-U6-shRNA and *BmSuc1*-PXL-U6-shRNA were kept in our laboratory. The ovary-derived cell line of *B. mori* (BmN) was stored in TC-100 insect culture medium containing 10% fetal bovine serum (ExCell, Shanghai, China) and 1% penicillin-streptomycin (Hyclone, Logan, UT, USA) at 27 °C.

### 4.2. Genotypic Identification

Gene sequences of normal *BmSpr* and *lem^l^* mutant *BmSpr* were obtained from NCBI (https://www.ncbi.nlm.nih.gov/, accessed on 5 October 2022) (Reference Sequence ID refer to Table 1). Since the *BmSpr* sequence in the *lem^l^* mutant has a continuous insertion of 27 nt compared with the normal type *BmSpr* sequence, the amplification product of the designed primers was 307 bp for the normal gene and 334 bp for the mutant gene. Genomic DNA was extracted from the legs of 36 pairs of male and female *lem^l^* moths according to the instructions of the Rapid Animal Genomic DNA Isolation Kit (Songon, Shanghai, China). After polymerase chain reaction (PCR) amplification, agarose gel electrophoresis (AGE) was used to detect the bands and determine the *lem^l^* mutant genotype according to the band size. To ensure the accuracy of the results, the target bands were further cut, recovered, and sequenced after AGE, followed by comparison with the sequence in the NCBI.

### 4.3. Extraction of Total Proteins and Enzymatic Activity Assay

Crude proteins were extracted from each group of larval tissues using a One-Step Animal Tissue Active Protein Extraction Kit (Sangon, Shanghai, China) following the manufacturer’s instructions. Each sample contained three independent individuals to eliminate any individual differences. The activity of BmSPR was determined as previously described [3]. The NOS enzyme activity, NO content, and PLA2 enzyme activity were determined according to the instructions of the Nitric Oxide Synthase (NOS) typed assay kit (Jiancheng, Nanjing, China), Nitric Oxide (NO) assay kit (Jiancheng, Nanjing, China), and Phospholipase A2 (PLA2) test kit (Lyell, Hefei, China), respectively.

### 4.4. Detection of Tyrosine Content

Briefly, 0.1 g of tissue powder to be measured was dissolved in 10 mL of 6 mol/L HCl and placed in a hydrolytic tube, into which N_2_ was filled; subsequently, the tube was sealed at 105 °C and dried for 24 h. The sample volume was fixed to 50 mL with ultrapure water, and 1 mL of the sample was freeze-dried for 2 days without light. Of 2 mL HCl dissolved sample, 1 mL was filtered into the sample bottle with a 0.22-μm disposable water membrane filter element. Samples were detected using an L-8900 automatic special amino acid analyzer, and three replicates were set in each group.

### 4.5. Microscopy

Second-instar a65 (+/+) and a65 (*lem^l^*/*lem^l^*) silkworms were dissected in PBS, and their fat bodies were taken separately for staining. Fat bodies were first fixed with 4% formaldehyde for 20 min at room temperature, and the tissue was then rinsed twice with PBS and incubated with 0.5 mg/mL Nile Red (Sigma, St. Louis, MI, USA) in a 1:1000 dilution of Nile Red and PBS for 30 min. After washing twice with PBS, the nucleus was treated with DAPI, and images were taken on an Olympus IX71 microscope powered by the cellSens Dimension software (Olympus, Tokyo, Japan). All images were imported and processed in Adobe Photoshop (Adobe, San Jose, CA, USA). The preparation of TEM and SEM samples was based on previous descriptions [34,35].

### 4.6. Identification of Mitochondrial and Cellular Damage

The crude protein of the second-instar wild-type silkworm and *lem^l^* homozygote silkworm was extracted as described in Section 2.3. Cytochrome C oxidase activity was determined as previously described [36]. The results were recorded, and the average value of three replicates was taken for each sample. Mitochondrial membrane potential analysis and mitochondrial ATP detection were performed according to the instructions of the Mitochondrial Membrane Potential Assay Kit with the JC-1 and ATP Assay Kit, respectively (Beyotime, Shanghai, China). JC-1 is an ideal fluorescent probe widely used to detect mitochondrial membrane potential. At low mitochondrial membrane potential, JC-1 is predominantly a monomer that yields green fluorescence with emission of 530 ± 15 nm. At high mitochondrial membrane potential, the dye aggregates yielding a red to orange colored emission (590 ± 17.5 nm). The levels of intracellular lipid oxidation and reactive oxygen species were determined according to the instructions of the Lipid Peroxidation MDA Assay Kit and the Reactive Oxygen Species Assay Kit, respectively (Beyotime, Shanghai, China).

### 4.7. Determination of BH4 and Superoxide Anion Content by HPLC

The level of BH4 was determined by an acid-base oxidation method, followed by fluorometric detection via HPLC. According to the previous method, total biopterin levels (BH4 + BH2 + biopterins) were determined by acid oxidation and BH2 + biopterin levels by basic oxidation [37]. The BH4 was determined as the difference between the areas under the curve in chromatograms for total biopterins and BH2 + biopterins [38]. The amount of processed cells taken for each test was 50 mg. In addition, measurement of 2-hydroxyethidium formation by HPLC was used to quantify superoxide production by methods adapted from those previously described [39,40].

### 4.8. RNA Isolation, cDNA Synthesis, and PCR

Total RNA was extracted from second-instar silkworm larvae using TRIzol reagent (Sangon, Shanghai, China) according to the manufacturer’s instructions. Subsequently, the RNA was treated with DNase I (TaKaRa, Dalian, China) to remove genomic DNA. For cDNA synthesis, 1 μg of total RNA was used in a ReverAid First Strand cDNA Synthesis Kit (Sangon, Shanghai, China) according to the manufacturer’s instruction. Reverse transcription PCR (RT-PCR) and quantitative real-time PCR (qRT-PCR) were performed to analyze the transcriptional levels of various genes in silkworm larvae. The RT-PCR cycling conditions were 95 °C for 5 min, followed by 29 cycles of 95 °C for 10 s, 55 °C for 15 s, and 72 °C for 30 s. The PCR products were separated by AGE on 1.0% agarose gels. The qRT-PCR was performed using power SYBR green PCR master mix (TaKaRa, Dalian, China) according to the manufacture’s recommendations, with denaturation at 95 °C for 5 min, followed by 40 cycles of 95 °C for 10 s, 51 °C/53 °C for 15 s, and 72 °C for 15 s in a CFX96 system (Bio-Rad, Hercules, CA, USA). The iQ5 software (Bio-Rad, USA) was used for qRT-PCR analysis. The *rp49* gene was used as an internal control. All samples were carried out in triplicate. The primers used in RT-PCR and qRT-PCR are listed in Table 2. Relative expression levels were calculated using the 2^−ΔΔCt^ method following the protocol of Livak and Schmittgen [41].

### 4.9. BmSpr Gene Silencing

The interference plasmid *BmSpr*-PXL-U6-shRNA was transfected into BmN cells using Effectene^®^ Transfection Reagent (Invitrogen, Shanghai, China) according to the manufacturer’s instruction. Three days after transfection, the cells were observed through red fluorescence under a fluorescence microscope (Olympus BX53, Tokyo, Japan) to confirm the transfection effect and cell status. Subsequently, a portion of the cells was collected for total RNA extraction to test the interference efficiency. All experiments were performed in three separate biological replicates.

### 4.10. Drug Addition to BmN Cells

The BmN cells were incubated with L-NAME, ISMN, and SOD at final concentrations of 0.1, 0.5, 1, 2, 5, and 10 mmol/L for 12, 24, or 48 h. Subsequently, the cells were collected for RNA extraction to detect cell proliferation to determine the optimal concentration and time of drug addition. All experiments were performed in three separate biological replicates.

### 4.11. Cell Proliferation Assay

According to the different experimental purposes, the BmN cells were treated via drug addition or gene interference, and the cell proliferation ability was detected to reflect the experimental effect. The cell proliferation ability was tested using the Cell Counting Kit-8 (Sangon, Shanghai, China) following the manufacturer’s instructions. All experiments were performed in three separate biological replicates.

### 4.12. DNA Fragmentation Detection

Genomic DNA of *B. mori* was extracted as described in Section 2.2. The dissolved products of the second-instar rearing silkworm were centrifuged at 13,000 r/min at 4 °C for 20 min to collect fragments of DNA. Subsequently, 0.2 mL of 6% perchloric acid (PCA) was treated at 4 °C for 30 min, followed by centrifugation at 13,000 r/min for 20 min at 4 °C. The precipitate was treated with 6% PCA 50 μL at 70 °C for 20 min, and 100 μL of diphenylamine solution was added; the mixture was stored at 30 °C overnight. The absorbance of intact DNA and fragment DNA at 600 nm was determined using a spectrophotometer (UV-9000, Shanghai, China). The assay method of the AGE was the same as that described elsewhere [42].

### 4.13. Bacteriostatic Test

The silkworms were washed thrice with 75% alcohol and homogenized in equal amounts. Subsequently, 50 μL aliquots of *Staphylococcus aureus* and *Escherichia coli* (cfu = 5 × 10^7^) were added, respectively, and the mixture was diluted 1000–10,000 times using normal saline (0.7%). After the addition of 200 μL of this mixture to agarose gel medium and incubation at 37 °C for 12 h, the results were observed. The fewer colonies had grown on the medium, the stronger the antibacterial ability of the corresponding silkworm’s body fluid was.

### 4.14. Statistical Analysis

All data were calculated as mean ± standard deviation. Differences between samples were analyzed by one-way analysis of variance (ANOVA). Means were compared by the least significant difference (LSD) tests. All statistical calculations were run using the DPS software (version 8.01) [43], and statistical significance was set at *p* < 0.05.

## Figures and Tables

**Table 1:** Information about the genes covered in this study.

Name	Reference Sequence ID	Protein ID
TAT	XM_021351181.2	XP_021206856.1
COX1	XM_038021628.1	XP_037877556.1
NOS1	NM_001043498.1	NP_001036963.1
NOS2	NM_001123336.1	NP_001116808.1
DDC	NM_001043709.1	NP_001037174.1
PPO	NM_001043870.1	NP_001037335.1
Moricin	NM_001043364.2	NP_001036829.2
Lebocin1	NM_001126260.2	NP_001119732.2
Enbocin1	NM_001044007.1	NP_001037472.1
Defensin	NM_001043905.2	NP_001037370.1
Enbocin2	NM_001098374.1	NP_001091844.1
Gloverin-2	NM_001044218.2	NP_001037683.1
Attacin1	NM_001043541.1	NP_001037006.1
Attacin2	S78369.1	AAB34519.1
CecropinA	NM_001043997.1	NP_001037462.1
CecropinD	NM_001043368.2	NP_001036833.1
Lysozyme	L37416.1	AAB40947.1
Apaf-1	NM_001200008.1	NP_001186937.1
Dronc	NM_001195467.1	NP_001182396.1
P53	NM_001177410.1	NP_001170881.1
Ice	NM_001043832.1	NP_001037297.1
Dredd	NM_001114865.2	NP_001108337.1
Suc1	NM_001126249.1	NP_001119721.1
Spr (+/+)	XM_004924359.4	XP_004924416.1
Spr (*lem^l^*/*lem^l^*)	AB465551.1	BAH56568.1

**Table 2:** Primers used in this study.

Primers	Forward Primers (5′ to 3′)	Reverse Primers (5′ to 3′)	Notes
NOS1	ATGGAAGTGCAATTCGAACA	TTAACTGCTCTGGCGATCTG	RT-PCR
NOS2	TGGCACATACCAACTAACC	AAACGCATACTGGAGACG	RT-PCR
q-Actin3	AGACGAGGCACAGAGCAA	TGTAGAAGGTATGATGCCAAA	RT-PCR
q-Spr	TAGACTTGAGTAAGGCATCG	TACGCCATTCCGCTCATA	RT-PCR
q-TAT	ATGGGTTGGATTGTC	TATCGTCGGTAAAGC	qRT-PCR
q-Cox1	TGCTGGAGGAGGAGACCC	GCTGAAGTAAAATATGCTCGTGT	qRT-PCR
q-rp49	CCCAACATTGGTTACGGTTC	GCTCTTTCCACGATCAGCTT	qRT-PCR
q-Moricin	TTAATGCTTTCTTTTCTTCGGTTT	TCATGTAGTACAGCCGCTCCA	qRT-PCR
q-Lebocin1	AGTTCTGGTGCTGTTCTT	CCATAGCGGTTCCTG	qRT-PCR
q-Enbocin1	TTTTCTTGTTCGTTGTTGTTTTCG	AGGTAGCTGCCGCCACCGTC	qRT-PCR
q-Defensin	AACCGTCTTTGACAACC	CGAACTCGCACCATAT	qRT-PCR
q-Enbocin2	TTTTCTTGTTCGTTGTTGTTTTCG	GCTGATGACGGCATCTCGC	qRT-PCR
q-Gloverin-2	CGGATCTCTGCTTGAAGACC	GCACTTTGGGACAAAACGAT	qRT-PCR
q-Attacin1	CTCGCTCTGGACAATGTAAACGG	CGCTCAGGTCGTGGTTGTTATT	qRT-PCR
q-Attacin2	CTCGCTCTGGACAATGTAA	CGCTCAGGTCGTGGTT	qRT-PCR
q-CecropinA	GGATTTCGCTTGCCCTATGA	AGCCCAGGTGGAAACTCTTC	qRT-PCR
q-CecropinD	ATTTTCGTTTTCGTGTTC	CTTTTGCCAGGGTGTC	qRT-PCR
q-Lysozyme	TTTCGCTTTGGTTGTCCTC	TCGCTGCCTTAGTAATGTCG	qRT-PCR
q-DDC	GCCTTGGACTGCGGTGATG	CTAGCCGTGCCCTGGATTA	qRT-PCR
q-PPO	TACTACGGCGACCTCCACAA	GACGGACGACACCCTGATG	qRT-PCR
q-Apaf-1	ACAGTTCACAACCCTCTAAAATCAC	GACTTTCTTACCACGCATCACC	qRT-PCR
q-Dronc	ACCCTGGAGCAGATGTCG	GGAGGTCCGTGAAGTTGG	qRT-PCR
q-P53	GGGCAATACAACTTCAGCG	CTTCTCGGCCTGGGACT	qRT-PCR
q-Ice	ATTCGCTGCCGACCAA	TTCGCACAGTGTCTGGATTA	qRT-PCR
q-Dredd	TAATAGTCGTTCTGACTTGGGACA	TCGGTATGCAATGCAGTTTCT	qRT-PCR

## Data Availability

The data presented in this study are available on request from the corresponding author.

## Supplementary Materials

The following supporting information can be downloaded at: https://www.mdpi.com/article/10.3390/ijms232012083/s1.
