# Supplementary material for: Exploring the Mystery of the Tetrahydrobiopterin Synthetic Defect Lethal Mutant leml from Birth to Death in the Silkworm Bombyx mori
Source: Int J Mol Sci. 2022 Oct 11;23(20):12083. doi: 10.3390/ijms232012083 (PMC9603568; doi:10.3390/ijms232012083)
Supplement: Supplementary file 1 [file ijms-23-12083-s001.zip › ijms-1953552-supplementary.pdf]

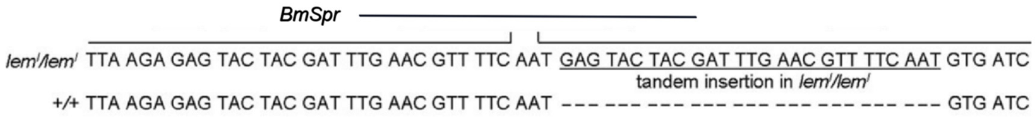

Figure S1. Differences in ORF sequences of *BmSpr* between +/+ and *lem^l/lem^l*.

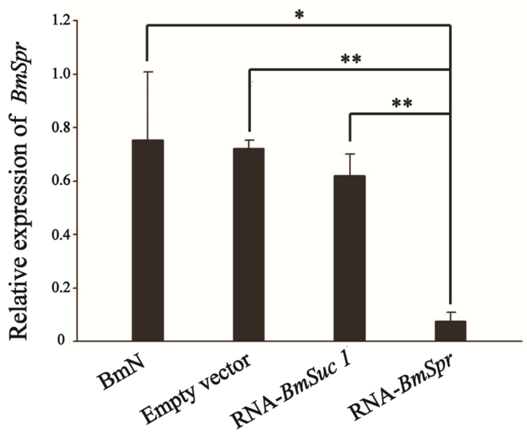

Figure S2. Interference efficiency verification after RNAi-*BmSpr*.

Data are mean values ± S.E.M (n = 3). Error bars represent mean ± SD; * $p < 0.05$; ** $p < 0.01$.

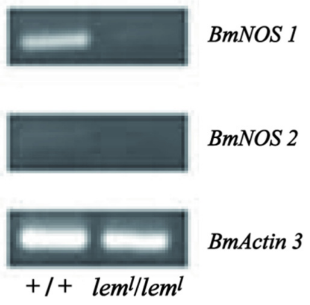

Figure S3. Expression analysis of *BmNOS* in second instar *lem^l* homozygous mutant. *BmActin3* is a reference gene.

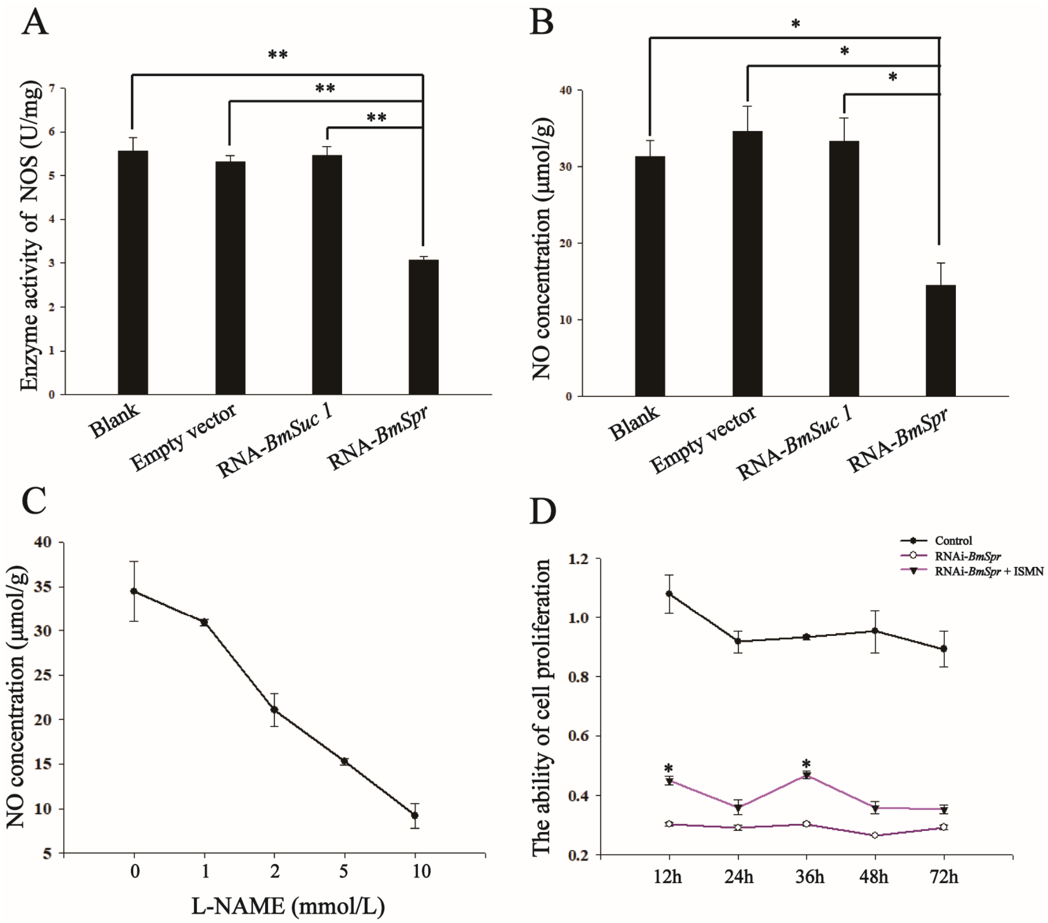

Figure S4. NOS uncoupling led to a reduced NO content after RNAi-*BmSpr* in BmN cells.

A. NOS enzyme activity decreased after RNAi-*BmSpr* in BmN cells. B. The content of NO decreased after RNAi-*BmSpr* in BmN cells. C. NO content decreased after adding L-NAME to BmN cells. D. The addition of ISMN can restore the ability of cell proliferation after RNAi-*BmSpr* in BmN cells. Absorbance was measured at 450 nm. The significance comparison objects in the figure were the RNAi-*BmSpr* group and the RNAi-*BmSpr* + ISMN group. The accession numbers of genes and proteins involved in the figure are shown in Table 2. Data are mean values ± S.E.M (n = 3). Error bars represent mean ± SD; * $p < 0.05$; ** $p < 0.01$; *** $p < 0.001$.

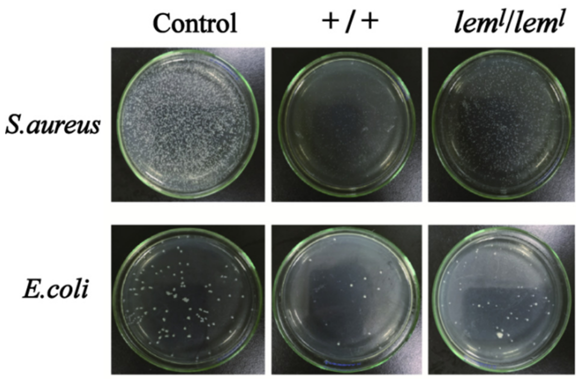

Figure S5. The humoral bacteriostatic level of second instar *lem^l* homozygous mutant decreased. The control was an equal volume of 0.7% normal saline.

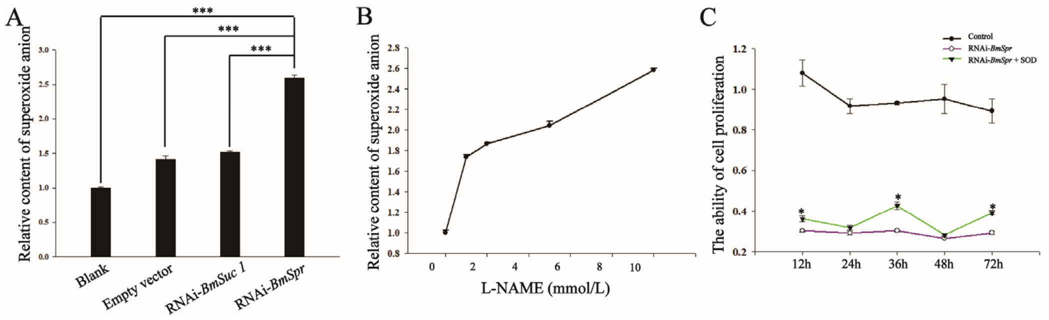

Figure S6. Increased O_2^- content could affect cell proliferation after RNAi-*BmSpr* in BmN cells.

A. O_2^- content increased after RNAi-*BmSpr* in BmN cells. B. O_2^- content increased after adding L-NAME to BmN cells. C. The addition of SOD can restore the ability of cell proliferation after RNAi-*BmSpr* in BmN cells. Absorbance was measured at 450 nm. The significance comparison objects in the figure are the RNAi-*BmSpr* group and the RNAi-*BmSpr* + SOD group. The accession numbers of genes and proteins involved in the figure are shown in Table 2. Data are mean values ± S.E.M (n = 3). Error bars represent mean ± SD; * $p < 0.05$; ** $p < 0.01$; *** $p < 0.001$.

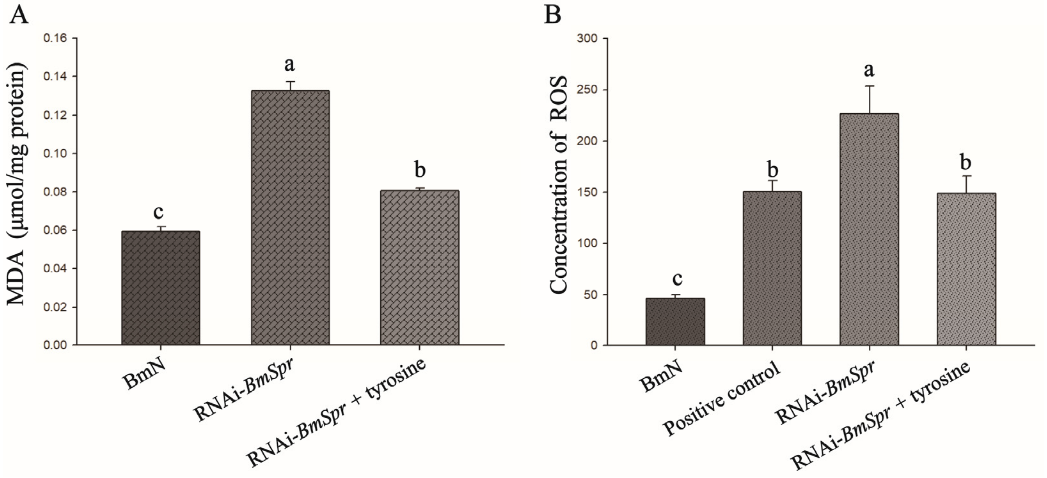

Figure S7. Contents of MDA (A) and ROS (B) in cells after torsion supplementation.

The positive control in figure B was the group in which the positive control reagent was added to normal BmN cells. The positive control reagent was a compound mixture called Rosup at a concentration of 50 mg/mL provided by the Reactive Oxygen Species Assay Kit. All values in the figure are mean ± SD. Bars labelled with different letters are significantly different (one-way ANOVA followed by LSD test, $p < 0.05$). ANOVA, analysis of variance; LSD, least significant difference.
